# Clinicopathological features and prognosis of mesenteric gastrointestinal stromal tumor: evaluation of a pooled case series

**DOI:** 10.18632/oncotarget.14880

**Published:** 2017-01-28

**Authors:** Fan Feng, Bin Feng, Shushang Liu, Zhen Liu, Guanghui Xu, Man Guo, Xiao Lian, Daiming Fan, Hongwei Zhang

**Affiliations:** ^1^ Division of Digestive Surgery, Xijing Hospital of Digestive Diseases, Fourth Military Medical University, Xi'an, Shaanxi, China

**Keywords:** gastrointestinal stromal tumor, mesentery, feature, prognosis

## Abstract

**Background:**

Due to the extremely rare incidence, data of clinicopathological features and prognosis of mesenteric gastrointestinal stromal tumors (GISTs) are limited. Therefore, the aim of the present study was to investigate the clinicopathological features and prognosis of mesenteric GISTs.

**Patients and Methods:**

Mesenteric GISTs cases were obtained from our center and from case reports and clinical series extracted from MEDLINE. Clinicopathological features and survivals were analyzed.

**Results:**

A total of 114 mesenteric GISTs were enrolled in present study. The most common symptom was abdominal pain (20/72, 27.8%), followed by abdominal mass (13/72, 18.1%) and distention (9/72, 12.5%). Most tumors exceeded 10 cm in diameter (71/112, 63.4%), exceeded 5/50HPF in mitotic index (50/85, 58.8%), and were high risk (82/90, 91.1%). The five-year disease free survival (DFS) and disease specific survival (DSS) was 57.7% and 60.1%, respectively. Tumor size and mitotic index were associated with DFS and DSS. The distribution of tumor size, histological type, mitotic index and NIH risk category were significantly different between mesenteric and gastric GISTs. Prognosis of mesenteric GISTs was worse than that of gastric GISTs. However, multivariate analysis showed that location was not an independent prognostic factor for mesenteric and gastric GISTs.

**Conclusions:**

Most mesenteric GISTs exceeded 10 cm in diameter, exceeded 5/50HPF in mitotic index and were high risk. Mesenteric GISTs differed significantly from gastric GISTs in respect to clinicopathologic features. Mitotic index and tumor size were prognostic factors for mesenteric GISTs. The prognosis were comparable between mesenteric and gastric GISTs.

## INTRODUCTION

Gastrointestinal stromal tumors (GISTs) are the commonest mesenchymal neoplasms of the gastrointestinal (GI) tract and represent 1-2% of all GI malignancies [1]. GISTs are considered to arise from the interstitial cells of Cajal (ICC) [2]. Most GISTs displayed spindle cell morphology (70%), followed by epithelioid (20%) and mixed phenotypes (10%) [3]. GISTs can occur anywhere throughout the GI tract and are seen most commonly in the stomach (40 to 70%) and small intestine (20 to 40%) [4]. GISTs that arise outside the GI tract as primary tumor are designated as extra-GISTs (EGISTs). EGISTs are located in the omentum, mesentery, liver, pancreas and retroperitoneum, etc [5].

Due to the extremely rare incidence, reporting of mesenteric GISTs has been limited to individual case reports and case series of small numbers. Studies involving large numbers of mesenteric GISTs are lacking. Thus, several questions remain unanswered, including clinical and pathological characteristics and prognosis. Therefore, the aim of the present study was to investigate the clinicopathological features and prognosis of mesenteric GISTs.

## RESULTS

The clinicopathological features were summarized in Table 1. There were 52 male (51.0%) and 50 female (49.0%). The median age was 57(6-84) years. The most common symptom was abdominal pain (20/72, 27.8%), followed by abdominal mass (13/72, 18.1%) and abdominal distension (9/72, 12.5%). One hundred and one patients underwent complete surgical resection (101/111, 91.0%), 3 patients underwent palliative resection (3/111, 2.7%), and 7 patients did not receive surgery (7/111, 6.3%).

**Table 1:** Clinicopathological characteristics of 114 cases of mesenteric GISTs

Characteristics	Parameters
Age (∑=113)	
≤60	68(60.2%)
>60	45(39.8%)
Gender (∑=102)	
Male	52(51.0%)
Female	50(49.0%)
Accompanied tumor (∑=44)	
GISTs with other locations	12(27.3%)
Other type of tumors	2(4.5%)
Symptoms (∑=72)	
Abdominal pain	20(27.8%)
Abdominal mass	13(18.1%)
Abdominal distension	9(12.5%)
Tumor size (∑=112)	
≤2cm	1(0.9%)
2.1-5cm	14(12.5%)
5.1-10cm	26(23.2%)
>10cm	71(63.4%)
Imaging features (∑=28)	
Solid	12(42.9%)
Cystic	3(10.7%)
Mixed	13(46.4%)
Surgical resection (∑=111)	
Complete resection	101(91.0%)
Incomplete resection	3(2.7%)
No surgery	7(6.3%)
Histological type (∑=90)	
Spindle	66(73.3%)
Epithelioid	13(14.5%)
Mixed	11(12.2%)
Mitotic index (∑=85)	
≤5	35(41.2%)
>5	50(58.8%)
Immunohistochemisty	
CD117(∑=50)	46(92.0%)
DOG-1(∑=11)	10(91.0%)
Mutational status (∑=18)	
KIT	5(27.8%)
PDGFRA	7(38.9%)
Wild type	6(33.3%)
NIH risk category (∑=90)	
Very low risk	1(1.1%)
Low risk	7(7.8%)
Intermediate risk	0
High risk	82(91.1%)
Adjuvant therapy (∑=41)	
Yes	25(61.0%)
No	16(39.0%)

GIST: gastrointestinal stromal tumor;

DOG-1: discovered on GIST 1;

KIT: c-kit proto-oncogene;

NIH: National Institutes of Health;

The tumors ranged from 0.4 to 39 cm (median, 12.0 cm; mean, 13.7 cm). Sixty-six patients displayed spindle cell morphology (66/90, 73.3%), 13 patients displayed epithelioid morphology (13/90, 14.5%) and 11 patients displayed mixed morphology (11/90, 12.2%). The mitotic index of 50 patients exceeded 5/50 HPF (50/85, 58.8%). CD117 positivity was detected in 46 patients (46/50, 92.0%), DOG-1 positivity was detected in 10 patients (10/11, 91.0%). Eighteen patients were analyzed for gene mutation status. Five patients carrying KIT mutation (5/18, 27.8%), 7 patients carrying PDGFRA mutation (7/18, 38.9%), the remaining 6 patients were wild type (6/18, 33.3%). According to NIH risk classification, 1 patient was very low risk (1/90, 1.1%), 7 patients were low risk (7/90, 7.8%), no patient was intermediate risk, 82 patients were high risk (82/90, 91.1%). Information of imatinib therapy were recorded in 41 patients, and 25 patients (61.0%) received imatinib therapy.

Survival data of 57 patients were eventually selected for analysis (Table 2). The follow up time ranged from 2 to 192 months (mean, 47.7 months; median, 27.0 months). Twenty-six patients showed recurrence or metastasis, 18 patients suffered from GIST related deaths. The 1-, 3- and 5-year DFS was 84.3%, 63.0% and 57.7%, respectively. The 1-, 3- and 5-year DSS was 92.3%, 67.5% and 60.1%, respectively. The DFS and DSS of mesenteric GISTs were shown in Figure 2.

**Table 2:** Survival data of 57 cases of mesenteric GISTs

Survival characteristics	Parameter
Follow up time	
Mean(m, ±SD)	47.7±48.6
Median(m, range)	27 (2, 192)
Survival data	
Recurrence or metastasis	26
GISTs related deaths	18
Survival rates (%)	
1-/3-/5-year DFS	84.3/63.0/57.7
1-/3-/5-year DSS	92.3/67.5/60.1

SD: Standard deviation;

DFS: disease-free survival;

DSS: disease-specific survival.

**Figure 1:**
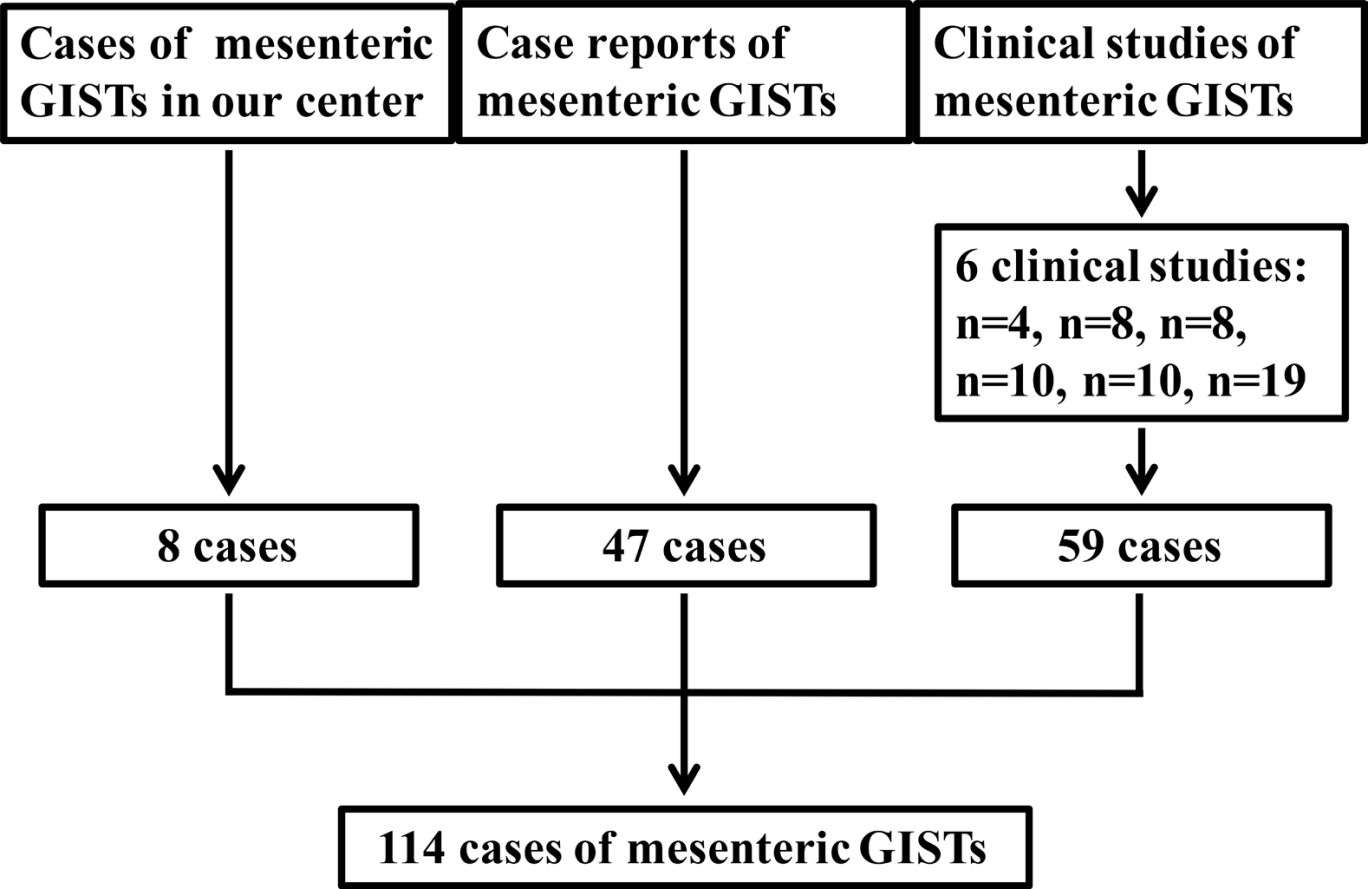
Schematic diagram regarding selection of mesenteric GISTs

**Figure 2:**
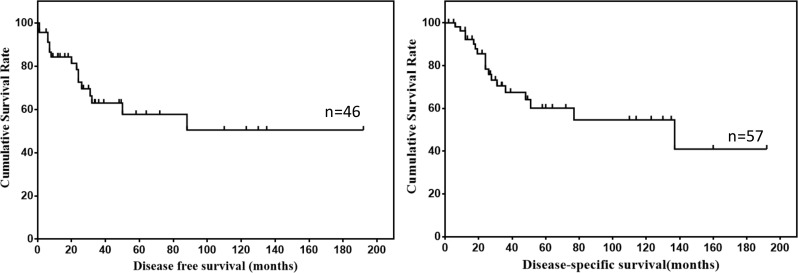
DFS and DSS of mesenteric GISTs

**Figure 3:**
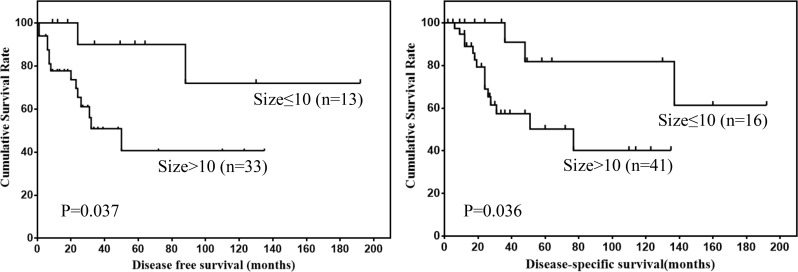
DFS and DSS of mesenteric GISTs by tumor size

**Figure 4:**
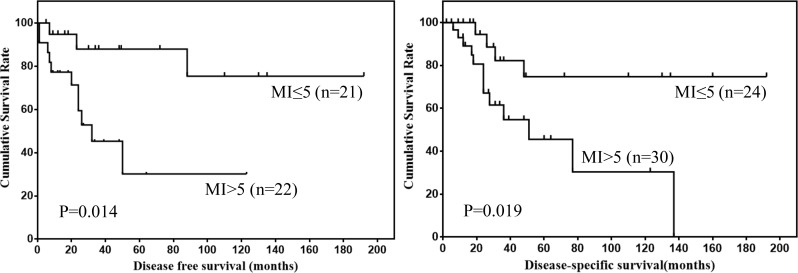
DFS and DSS of mesenteric GISTs by mitotic index

Prognostic factors for DFS and DSS of mesenteric GISTs were shown in Table 3. Univariate analysis showed that tumor size and mitotic index were prognostic factors for mesenteric GISTs. However, multivariate analysis showed that tumor size was the only independent risk factor for DSS. The DFS and DSS of mesenteric GISTs according to tumor size and mitotic index were shown in Figure 3 and 4.

**Table 3:** Prognostic factors for DFS and DSS in patients with mesenteric GISTs according to univariate and multivariate analysis (*n* = 57)

	Univariate analysis			Multivariate analysis		
Prognostic factors	β	Hazard ratio (95% CI)	*P* value	β	Hazard ratio (95% CI)	*P* value
DFS						
Tumor size (≤10/>10)	2.178	8.825 (1.138-68.438)	0.037			
Mitotic index (≤5/>5)	1.661	5.263 (1.398-19.804)	0.014	1.216	3.374 (0.864-13.181)	0.080
NIH risk category (1,2,3/4)	1.740	5.700 (0.388-83.746)	0.204			
DSS						
Tumor size (≤10/>10)	1.587	4.888 (1.108-21.566)	0.036	2.104	8.197 (1.042-64.498)	0.046
Mitotic index (≤5/>5)	1.358	3.890 (1.248-12.128)	0.019			
NIH risk category (1,2,3/4)	3.541	31.543 (0.160-6199.591)	0.200			

DFS: disease-free survival;

DSS: disease-specific survival;

NIH: National Institutes of Health.

CI: Confidence interval.

The clinicopathological features of 114 mesenteric GISTs including age, gender, tumor size, histological type, mitotic index and NIH risk category were compared with 297 gastric GISTs in our institution (Table 4). The results showed that the distribution of tumor size, histological type, mitotic index and NIH risk category were significantly different between mesenteric and gastric GISTs (all *P* < 0.05).

**Table 4:** Comparison of selected clinicopathological parameters between mesenteric and gastric GISTs

Characteristics	Mesentery(*n*= 114)	Stomach(*n*= 297)	*P* value
Age			
≤60	68	168	0.576
>60	45	129	
Gender			
Male	52	155	0.907
Female	50	142	
Tumor size			
≤2cm	1	96	<0.001
2.1-5cm	14	107	
5.1-10cm	26	72	
>10cm	71	22	
Histological type			
Spindle	66	275	<0.001
Epithelioid	13	3	
Mixed	11	19	
Mitotic index			
≤5	35	163	0.027
>5	50	134	
NIH risk category			
Very low	1	83	<0.001
Low	7	58	
Intermediate	0	87	
High	82	69	

NIH: National Institutes of Health.

**Figure 5:**
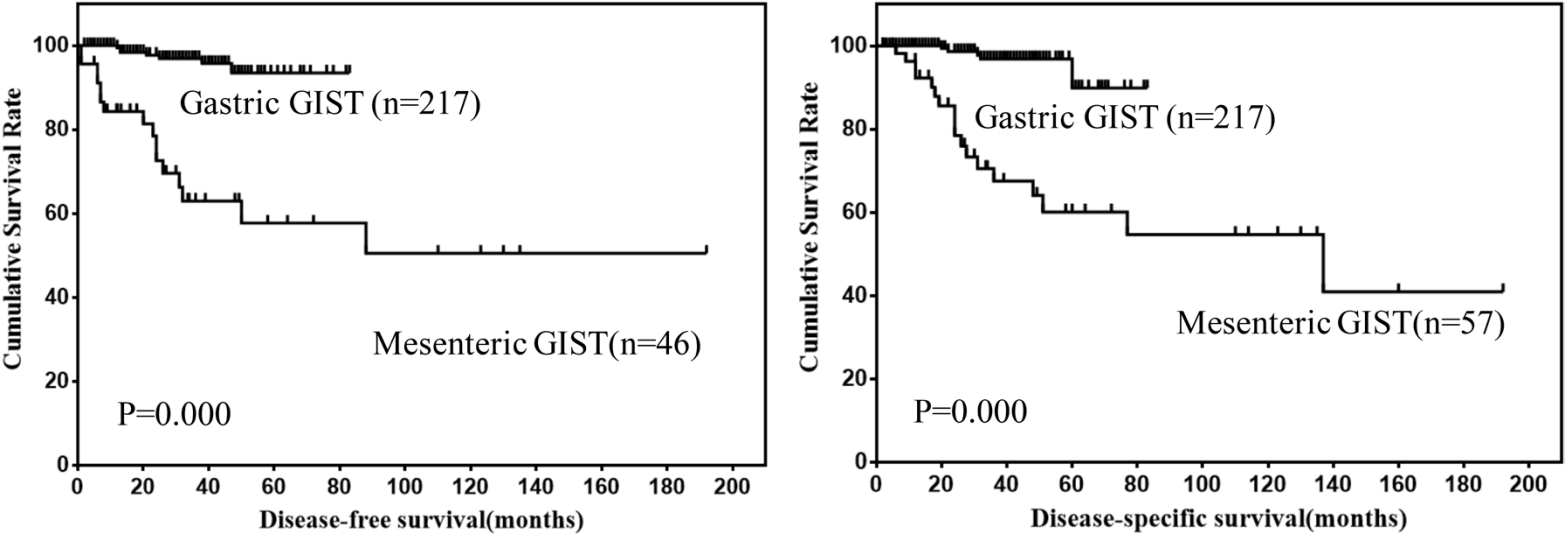
Comparison of DFS and DSS between mesenteric and gastric GISTs

In order to compare the prognosis of mesenteric GISTs with gastric GISTs, 57 cases of mesenteric GISTs and 217 cases of gastric GISTs with follow up data were analyzed. The results showed that DFS and DSS of mesenteric GISTs were significantly lower than that of gastric GISTs (Figure 5). Further, multivariate analysis was performed to evaluate the prognostic value of locations (Table 5). However, the results showed that location was not an independent risk factor for prognosis of mesenteric and gastric GISTs (both *P* > 0.05).

**Table 5:** Comparative survival analysis of mesenteric and gastric GISTs using univariate and multivariate analysis

Survival	Mesentery	Stomach	Univariate analysis			Multivariate analysis		
	(*n*= 57)	(*n*= 217)	β	HR (95% CI)	*P*	β	HR (95% CI)	*P*
DFS								
1-year	84.3	99.5	2.331	10.293 (4.184-25.320)	0.000	0.702	2.018 (0.474-8.581)	0.342
3-year	63.0	96.9						
5-year	57.7	93.5						
DSS								
1-year	92.3	100.0	2.438	11.451 (4.174-31.417)	0.000	0.723	2.060 (0.542-7.822)	0.289
3-year	67.5	96.8						
5-year	60.1	89.9						

DFS: disease-free survival;

DSS: disease-specific survival;

CI: confidence interval;

HR: hazard ratio.

## DISCUSSION

Due to the extremely rare incidence, studies involving large numbers of mesenteric GISTs are lacking. Therefore, the aim of the present study was to investigate the clinicopathological features and prognosis of mesenteric GISTs. The present study represents the largest analysis of mesenteric GISTs.

The precise etiology of mesenteric GISTs remains to be clarified. Some investigators have proposed that EGISTs are mural tumors with extensive extramural growth resulting in eventual loss of connection with gut wall [6]. However, this hypothesis lacks evidence. On the other hand, other researchers have proposed that GISTs may arise from a common precursor cell of ICC and smooth muscle cell, which may account for their growth from and outside the GI tract [7]. Terada et al. have demonstrated the existence of scattered KIT-positive ICC like cells in surface of the normal mesocolon [8]. This further provide evidence for the hypothesis that EGISTs may arise from precursor cell of ICC outside the GI tract. However, the existence of ICC like cells in the mesostenium have not been identified.

The spectrum of clinical presentation of GISTs is broad and depends on tumor location and tumor size. For mesenteric GISTs, tumors appear to have enough space to grow and may present clinical symptoms after a significant period of time with a considerable tumor size. In our present study, most tumors exceeded 10 cm in diameter. Thus, early diagnosis of mesenteric GISTs is very difficult. Once mesenteric GISTs reached a significant size, symptoms will appear. In our present study, the most common symptoms include abdominal pain, mass and distension.

Even with R0 resection, there is a high risk of recurrence and distant metastasis. However, no mention of mesenteric GISTs specific recurrence or metastasis was made previously. In our present study, half of patients with tumor progression after R0 resection suffered from abdominal recurrence. For distant metastasis, the most common site was liver. Metastasis to lung and brain was also occasionally found.

In 1998, Hirota et al. reported their groundbreaking discovery of KIT mutations in GISTs. It is now established that 70% to 80% of GISTs harbor KIT mutation [9], and PDGFRA mutation occur in approximately 8% to 10% of GISTs [10]. In our present study, gene mutations were recorded in only eighteen patients. Among them, 7 patients (38.9%) carrying PDGFRA mutation. The incidence of PDGFRA mutation in our present study was relatively higher than previous report. This indicated that the incidence of KIT and PDGFRA gene mutation could be various from each other depend on the location of GISTs. However, the association between the tumor location and gene mutation status needs further investigation.

In our present study, most tumors exceeded 10 cm in diameter and almost all the tumors were high risk. Therefore, the existing classification criteria which defined by a combination of mitotic index and tumor size may not be applicable to mesenteric GISTs. Reith et al. reported that high cellularity, mitotic index exceeds 2/50 HPF and presence of necrosis were factors indicative of a potentially aggressive clinical course for EGIST [11]. However, the relatively short follow up period in the study may result in bias of the data. Thus, a more appropriate grading system may be needed for the classification of EGISTs.

Tumor location is also one prognostic factor for GISTs [12], and it was considered that EGISTs were more aggressive than gastric GISTs. However, the modified NIH risk classification distinguishes only gastric from non-gastric GISTs, and the prognosis of mesenteric GISTs are not discussed. Thus, the prognosis of mesenteric and gastric GISTs were compared. We found that the prognosis of mesenteric GISTs was significantly worse than that of gastric GISTs. However, multivariate analysis showed that tumor location was not an independent risk factor for prognosis of mesenteric and gastric GISTs. We considered that the poor prognosis of mesenteric GISTs was mainly attributed to the larger tumor size and higher mitotic index, not to location.

There were a few limitations in our present study. Firstly, the present study is a retrospective analysis and the completeness of data is limited. Secondly, the sample size was not large enough, which will result in statistical bias. Thirdly, the clinicopathological features and prognosis of mesenteric GISTs were not compared with EGISTs in other locations.

## CONCLUSIONS

Most mesenteric GISTs exceeded 10 cm in diameter, exceeded 5/50HPF in mitotic index and were high risk. Mesenteric GISTs differ significantly from gastric GISTs in respect to clinicopathologic features. Mitotic index and tumor size were risk factors for prognosis of mesenteric GISTs. The prognosis were comparable between mesenteric and gastric GISTs.

## PATIENTS AND METHODS

GISTs cases of the mesentery were from our institution and literature. From May 2010 to March 2015, 8 cases of mesenteric GISTs were diagnosed and treated in our institution. Literature search of MEDLINE was performed for all articles in English published from 1999 through 2015. MEDLINE search resulted in 36 case reports [7, 8, 13–46] including 47 cases and 6 case series [47–52] including 59 cases. As a result, a total of 114 mesenteric GISTs patients were identified (Figure 1). In addition, the clinicopathological characteristics of 297 patients of gastric GISTs in our center were analyzed and compared with mesenteric GISTs. Among them, the prognosis of 217 gastric GISTs patients with follow-up data were analyzed and compared with mesenteric GISTs. This study was approved by the Ethics Committee of Xijing Hospital, and written informed consent was obtained from the eight patients in our center.

Data including age, gender, accompanied tumor, symptoms, tumor size, imaging features, surgical intervention, histological type, immunohistochemical features, mutational status, mitotic index, NIH risk category, adjuvant therapy, tumor progression and survival data were recorded. The tumors were categorized into very low, low, intermediate and high risk groups according to the modified NIH risk classification criteria [53]. For survival analysis, the inclusion criteria were listed as follows: 1. without distant metastasis, 2. without GISTs in other locations, 3. R0 resection, 4. without other malignant tumors, 5. without neoadjuvant imatinib therapy, 6. with follow up data. Due to data acquisition, completeness of data is limited.

Data were processed using SPSS 22.0 for Windows (SPSS Inc., Chicago, IL, USA). Discrete variables were analyzed using the Chi-square test or Fisher's exact test. Numerical variables were expressed as the mean ± SD unless. Significant predictors for prognosis identified by univariate analysis were further assessed by multivariate analysis using Cox's proportional hazards regression model was employed for multivariate analysis. Evaluation of disease-free-survival (DFS) and disease-specific-survival (DSS) were obtained by the Kaplan-Meier method. DFS was defined as the length of time from the date of surgery to the date of recurrence. DSS was defined as the length of time from the date of surgery to the date of cancer associated death. The P value was considered to be statistically significant at the 5% level.

## Abbreviations

GIST: Gastrointestinal stromal tumor
HPF: High power field
DFS: Disease-free survival
DSS: Disease-specific survival
NIH: National Institutes of Health
GI: Gastrointestinal
ICC: Interstitial cells of Cajal
EGIST: Extra-gastrointestinal stromal tumor
DOG-1: Discovered on GIST 1
KIT: c-kit proto-oncogene protein
SD: Standard deviation
CI: Confidence interval
HR: Hazard ratio.
